# Supplementary material for: Application of elementary probability models for text homogeneity and segmentation: A case study of Bible
Source: PLoS One. 2024 Jun 7;19(6):e0303432. doi: 10.1371/journal.pone.0303432 (PMC11161094; doi:10.1371/journal.pone.0303432)
Supplement: Attachment — Submitted filename: report020502.pdf [file pone.0303432.s001.pdf]

Dear Editor,

The article "Homogeneity and change point detection using forward and backward processes: for Bible texts" sets out to analyze the Bible in Tigrigna, Amharic and English languages, by forward/backward processes, in the framework of some well-known statistical models (Zipf, Heaps and Mandelbrot laws). It is found that, the Bible is a heterogeneous concatenation of 66 books for each of the three languages with relatively low error rate.

In my opinion this paper deals with an interesting problem and seems to be technically correct. The author has also tried to express the subject clearly with the help of figures and tables. However, there are some serious concerns about this version of the manuscript. I strongly suggest author to revise the paper according to following points before publication.

Warm regards,
Ali Mehri

Minor points:

1- The manuscript needed to be reread since there is a considerable number of typos and incorrect grammar throughout the text. I list some of the language problems in the following:

- Abstract

  Zifp-Mandelbrot --> Zipf-Mandelbrot

- Introduction

  therefore,expected --> therefore, expected

  Bahdur --> Bahadur

  mendelbrot --> Mandelbrot

  homogeniety --> homogeneity

- Theoretical Background

  Hurvitz zeta function --> Hurwitz zeta function

  number of word --> number of words

- Bible Classification

  old testament --> Old Testament

  (Job --> (Job

  new Testament --> New Testament

- Application to Bible Books in 3 Languages

  more then 600 words --> more than 600 words

2- Citations in the text are started from [16]. They should be sorted in ascending order: [1], [2], [3], … .

3- Text should be justified.

4- What is [?] in lines 11 and 12 of page 2/8.

5- All formulas should be labeled by numbers.

6- All parameters and variables in formulas have to be introduced in the text.

7- P.5/8, L.84 & L.85: Short paragraphs and unintelligible sentences.

8- It will be illustrative if author give an example for calculation of parameters in table 3.

9- Fig.4 and Fig.5 should be explained clearly in the text. Moreover, red and blue colors have to be indicated in fig.4 and fig.5.

10- All references have to be written in the same style. Please take a look at references 8, 9, 11, …. In some references authors name is written completely and in some others just the first letter of their name written.

Major concern:

1- P.4/8, L.47: Author has to explain Table (and the next tables) completely. Its all parameters and values should be clarified in detail.

2- Heat maps are not properly described. Author has to clearly explain what they refer to.

3- All parameters in tables have to be determined clearly in the captions.

4- P.6/8, L.96: Table does not have caption! What is the third column in this table?

5- Please explain why “longer texts consistently have lower error rates”.

6- Forward and backward processes should be described in detail.
