# Supplementary material for: Application of elementary probability models for text homogeneity and segmentation: A case study of Bible
Source: PLoS One. 2024 Jun 7;19(6):e0303432. doi: 10.1371/journal.pone.0303432 (PMC11161094; doi:10.1371/journal.pone.0303432)
Supplement: Attachment — Submitted filename: Review_Response_To_PLOS_ONE_Journal.pdf [file pone.0303432.s002.pdf]

Responses to Reviewers' Comments for Manuscript PONE-D-23-20994

Homogeneity and Change Point Detection Using Forward and Backward Processes: For Bible Texts

Addressed Comments for Publication to

PLOS ONE

by

Berhane Abebe

Dear Roy Cerqueti, Ph.D.,

Please find enclosed the revised version of my previous submission entitled "Homogeneity and Change Point Detection Using Forward and Backward Processes: For Bible Texts" with manuscript number PONE-D-23-20994. We would like to thank you and the reviewers for the valuable comments which help improving the quality of my manuscript. In this revision, I have carefully addressed the reviewers' comments and made one major modification in the analysis of text segmentation. A summary of main modifications and a detailed point-by-point response to the comments from Reviewers 1 and 2 are given below.

Sincerely,

Berhane Abebe

Authors' Response to the Editor

General Comments. The paper has an interesting research question, but it it not publishable in its current form. The reviewers have some doubts about the effectiveness of a revision round, but I want to give you the opportunity to revise deeply the paper.

Response: I really appreciate your handling of the review process.

Aside from the reviewers' suggestions on typos and grammatical issues, which I thoroughly fixed, I verified the document and addressed all of them as follows::

1. The findings were thoroughly discussed.
2. Instead of the pairwise error % for the 66 books of the Bible, text segmentation of one genre of the English edition was added.
3. New figures have been uploaded, and the paper has been revised to meet the journal's criteria as much as feasible.

Authors' Response to Reviewer 1

General Comments. General comment about the work.

Response: Thank you for your feedback.

We have carefully addressed all the issues item by item as follows.

Comment 1

there is a considerable number of typos and incorrect grammar throughout the text.

Response: Thank you for the comment.

I have carefully revised the whole manuscript and addressed almost all typos and grammatical errors.

Comment 2

All tables should be explained along with its values and parameters.

Response: Thank you for the comment.

I totally agree. I have also added new tables and deleted some unnecessary ones, (duplicated analysis) in the new manuscript.

Statistical text segmentation was included for one heterogeneous genre rather than repeating error percentage estimation utilizing 66 books.

Comment 3

Please explain why “longer texts consistently have lower error rates”.

Response: Thank you for the comment.

I do agree, But due to change of analysis to text segmentation rather than error estimation the statement was removed along with the results.

Comment 4

Forward and backward processes should be described in detail..

Response: Thank you for the comment.

A definition of the processes was provided, along with an example.

Authors' Response to Reviewer 2

General Comments. The premises and the initial idea of the paper are interesting, but the part of the empirical analysis is meagre and needs to be completely argued

Response: Thank you for your feedback.

In the current version of the book, I have given a detailed explanation for the empirical analysis.

Comment 1

Methodology section is also a simple list of formulas left somewhat to itself and is quite unrelated from the empirical analysis.

Response: Thank you for your feedback.

The theoretical section has been renamed methodology and updated in a more intelligible manner where they have been applied.

Comment 2

The title should be made more intriguing.

Response: Thank you for the comment.

I agree that the title should be more appealing. As a result, I've made a change in the current version of the manuscript.

Comment 3

Beware of copy and paste, figures and their order.

Response: Thank you again

In the revised version of the manuscript, care has been taken.
