# Supplementary material for: Application of elementary probability models for text homogeneity and segmentation: A case study of Bible
Source: PLoS One. 2024 Jun 7;19(6):e0303432. doi: 10.1371/journal.pone.0303432 (PMC11161094; doi:10.1371/journal.pone.0303432)
Supplement: Attachment — Submitted filename: Response to Reviewers.pdf [file pone.0303432.s003.pdf]

Responses to Reviewers' Comments for Manuscript PONE-D-23-20994

Application of elementary probability models for text homogeneity and segmentation: a case study of Bible

Addressed Comments for Publication to

PLOS ONE

by

Berhane Abebe

Dear Roy Cerqueti, Ph.D.,

Please find enclosed the revised version of my previous submission entitled "Application of elementary probability models for text homogeneity and segmentation: a case study of Bible" with manuscript number PONE-D-23-20994. I would like to thank you and the reviewers again for the valuable comments which help improving the quality of my manuscript. In this revision, I have carefully addressed the Editor's comments and and journal Requirements. A summary of main modifications and a point-by-point response to the comments from Editor's are given below.

Sincerely,

Berhane Abebe

Authors' Response to the Editor

General Comments. In order to meet PLOS ONE publication criteria please ensure the manuscript has been copy edited.

Response: I really appreciate your handling of the review process.

Based on the Editor's suggestions on typos and grammatical issues, which I thoroughly fixed, I verified the document and addressed all of them as follows::

Comment 1

Please include supporting citations for lines 171-176.

Response: Thank you for the comment.

I do agree. I have included the relevant citations.

Comment 2

Typo on line 244.

Response: Thank you for the comment.

I totally agree. books were written as bookks and corrected.

Comment 3

Grammatical error in line 247.

Response: Thank you for the comment.

I do agree, "All Bible translations results under investigations are statistically valid" has been replaced by "All the Bible translations under investigations are statistically valid"

General Comments. Any changes to the reference list should be mentioned in the rebuttal letter .

Response: Thank You for the comment.

After carefully going through the reference materials, I have eliminated several of the unused ones that were mentioned in the .bib file and updated the remaining ones, which are shown below.

1. In reference No. 6 publisher added
2. In reference No. 12 publisher name Routledge was replaced by Taylor & Francis
3. In reference No. 14 @incollection was updated to @article
4. In reference No. 15 @article was updated to @inproceedings
